# Arsenic in Chorea

**Published:** 1880-07

**Authors:** 


Arsenic in Chorea.—According to *Buchardat's Ann.* for 1880, we find that, from observations made in the service of M. Siredey, by M. Pomel, upon the treatment of chorea, the following deductions are made:

1st. Of all the remedies employed in chorea, those which are the most rapid and sure are the arsenical preparations; particularly arsenious acid, which at once produces rapid amelioration of the symptoms, and brings about a speedy cure.

2. Grave cases of chorea that have resisted other treatment yield, frequently with promptness, to arsenic.

3. To obtain the full benefit of the arsenical treatment, it is necessary to administer the medicine in such doses as to produce, as speedily as possible, the constitutional effects or signs of arsenical saturation.

4. Even in children affected with chorea, no hesitation should be felt in giving strong doses of arsenic, in order to reach the point of saturation quickly.

5. Without denying the possibility of danger in the use of arsenic in the treatment of chorea, yet no case has thus far been recorded tc establish the fact.

The preparation used by M. Siredey is Boudin's solution, containing 1 milligram (1-65th of a grain) of arsenious acid to 1 gram (15 grains) of solvent.

M. Sireley commences, in the case of a young and moderately vigorous subject, with ten grams of the solution a day, well diluted, and increases the dose each day by five grams. This dose is first diluted and then taken in divided doses during the day. Should vomiting or nausea occur the quantity must be diminished For a child of eight or ten years, from two to four grams of the solution will be sufficient to commence with, increasing it daily by two grams.

*Note by Translator.*—As the solvent in Boudin's preparation is not given, Fowler's solution may be substituted. Its equivalent dose would be twenty minims daily, increased in the above proportion.

